# Recovery From Ostracism Distress: The Role of Attribution

**DOI:** 10.3389/fpsyg.2022.899564

**Published:** 2022-05-11

**Authors:** Erez Yaakobi

**Affiliations:** Ono Academic College, Kiryat Ono, Israel

**Keywords:** social exclusion, ostracism, attribution, intervention, death anxiety

## Abstract

Ostracism is known to cause psychological distress. Thus, defining the factors that can lead to recovery or diminish these negative effects is crucial. Three experiments examined whether suggesting the possible causes of ostracism to victims could decrease or eliminate their ostracism distress. They also examined whether death-anxiety mediated the association between the suggested possible cause for being ostracized and recovery. Participants (*N* = 656) were randomly assigned to six experimental and control groups and were either ostracized or included in a game of Cyberball. Two control conditions were used: participants who were ostracized but received no explanation and participants who were included. Immediately after the ostracism experience, participants in the experimental groups were presented with one of four causes for being ostracized, using locus of control (internal, external) and stability (stable, unstable), the two causal dimensions of Weiner’s attribution theory. After a short delay they were administered a mood or needs-satisfaction questionnaire. The results highlight the interaction between locus of control and stability, and underscore the relative importance of different attributions in alleviating self-reported ostracism distress. Specifically, both external and unstable attributions decreased distress, and an unstable attribution led to complete recovery in some participants. Thus, recovery from ostracism may be accelerated when the victim receives an explanation for ostracism that attributes the incident to unstable, external causes soon after the incident. Death-anxiety fully mediated the association between locus of control attribution and mood, but for on needs-satisfaction or the stability of the attribution.

## Introduction

Ostracism effects are widespread in the workplace, school, family, the military, religious groups, and organizations (e.g., Williams, 2007; Sommer and Yoon, 2013; Chung and Kim, 2017; Zhang et al., 2017; Wesselmann et al., 2018). Numerous studies indicate that ostracism occurs in the virtual realm as well [e.g., Donate et al., 2017; see meta-analysis by Hartgerink et al. (2015)].

The experience of ostracism has negative effects on both immediate (reflexive) and delayed (reflective) physiological, cognitive, affective, motivational, and behavioral outcomes, offline and online (e.g., Williams, 2009; Kouchaki and Wareham, 2015; Buelow and Wirth, 2017). For example, ostracized individuals’ subsequent behavior was reported to include greater risk-taking (e.g., Buelow and Wirth, 2017), aggression (Liu et al., 2018), dishonesty (Kouchaki and Wareham, 2015), racist attitudes (Bernstein et al., 2014), and less prosocial behavior (Twenge et al., 2007). Given these negative effects, it is crucial to better understand how to alleviate the distress underlying these outcomes.

One of the coping mechanisms that can alleviate distress is to attribute the cause of ostracism to specific factors. Snoek (1962) argued that attribution is one of the key cognitive processes that occur in the reflective stage. More recently, Williams (2009) has suggested that attributions could alleviate the effects of ostracism. Goodwin et al. (2010) examined whether attributing ostracism to racial prejudice mediated recovery. They indicated that Whites attributed ostracism to racism when the other players were Black; by contrast, Blacks attributed ostracism to racism when the other players were White or Black. Within a few minutes after attribution, the participants reported feeling less distress, but attributing ostracism to racial prejudice impeded their recovery. However, these authors did not characterize the types of attribution used by the ostracism victims. Tuscherer et al. (2016) found that the perceived fairness of being ostracized moderated victims’ ostracism response, but they did not systematically vary fair and unfair attributions. Bernstein et al. (2018) examined people’s responses to others’ exclusion experiences and found that internal attributions decreased the desire for affiliation to a greater extent than external or ambiguous attributions. They also found that empathy toward the target mediated this association. However, people’s responses to others’ exclusion experiences cannot be deduced from the effects of attribution on the ostracized victims. Moreover, Bernstein et al. only examined the locus of causality, but did not examine other attribution factors. Recently, Yaakobi (2022) have found that attachment orientation mediates the relationship between locus of attribution and ostracism distress. Yaakobi (2021) found that both locus of control (henceforth: locus) and stability attribution are associated with immediate ostracism distress. However, neither of these studies examined the temporal effects of the interaction of locus and stability on recovery after a short delay or the possible mediation processes underlying these effects. Thus, to date, there has been no empirical examination of the role of different types of attribution in alleviating or eliminating victims’ ostracism distress, specifically in the reflective stage following the ostracism event or its underlying mechanism.

The three experiments reported below were designed to fill this gap by examining how recovery might be affected by an intervention in which ostracism victims receive explicit cues that prompt specific types of attributions for the ostracism incident. These were examined in the reflective stage *after* victims had the opportunity to dwell on their ostracism experience and its possible causes, which often includes a search for attribution as a coping mechanism. The possible mediation of death anxiety on these effects was also examined. These experiments thus respond to the call for more research “to determine the recovery rate as a function of the attributed ostracism motive” (Williams, 2009, p. 296; Park et al., 2017). Weiner’s well-established attribution theory (1972, 1985) served as the foundation for developing the principles of the attribution intervention.

### Attribution Theory

Attributions are defined as causal explanations people construct to interpret their world and adapt to their surroundings. They are crucial when individuals find themselves in new, important, as well as negative situations (Weiner, 1985). According to the attributional theory of motivation (Weiner, 1972, 1985), all perceived attributions of success and failure have three causal dimensions: locus (external vs. internal), stability (unstable vs. stable), and controllability (controllable vs. uncontrollable). Here the focus was on locus and stability. This is because in Williams’ (2009) need-threat model, sense of control is considered to be one of the basic needs. Including controllability could thus lead to methodological biases which would be even more problematic when examined in parallel with manipulating locus and stability.

Internal attribution refers to the belief that an event was caused by internal factors such as a person’s intelligence, whereas external attribution refers to the belief that an event was caused by external factors such as luck. Stable attribution refers to belief that the cause of an event cannot change over time (e.g., personality) while unstable attribution relates to causes that can change over time (e.g., effort). The four possible combinations of internal–external and stable–unstable causes yield four types of attributions that people formulate to explain their own actions and those of others.

For example, a victim of ostracism may attribute the incident to a lack of effort on her part in a game, which corresponds to an internal, unstable (temporary) attribution. Alternatively, the victim may feel that she was ostracized because of task difficulty, which assigns attribution to an external, stable factor. A victim who believes that she was ostracized because she was simply unlucky is making an external, unstable attribution. A victim who believes that she was ostracized because people do not like her personality is making an internal, stable attribution. In this study, it was hypothesized that some forms of attribution could alleviate or even eliminate the effects of ostracism on needs-satisfaction and mood to an extent comparable to participants who were not ostracized. More generally, the aim was to explore how victims’ cognitive interpretations of the causes of ostracism were associated with the dynamics of their resultant emotions, thus shedding light on the possible underlying links between attributions and ostracism distress.

### Mediating Role of Death-Anxiety

Numerous studies have shown that the feeling of being included in social networks is a core individual need (Case and Williams, 2004). Terror Management Theory (TMT, Greenberg et al., 1997; see meta-analysis in Burke et al., 2010) has been posited to be linked to ostracism in that death anxiety may prompt similar effects to those elicited by ostracism (Case and Williams, 2004), since the ostracism experience is perceived as the negation of other’s existence. Individuals need to be recognized as sentient humans to be shielded against a sense of angst and purposelessness (Greenberg et al., 1997; Solomon et al., 2004). The key tenet of TMT is that individuals who feel they can contribute in a meaningful way to the world are protected from thoughts of death. When people feel they are being valued in their culture, they feel that their legacy will make an impact beyond their death and that their achievements will be remembered (Greenberg et al., 1997; Solomon et al., 2004). By extension, individuals who are rejected by their culture are more likely to feel that they are not of value and may thus experience greater death anxiety (Becker, 1971). Thus, when this need is not met, it may threaten existentialistic concerns. Case and Williams (2004) suggested that similar to other mortality salience inductions, ostracism may trigger defenses based on individual’s cultural worldview. Previous studies have revealed that death-anxiety mediates ostracism distress (Yaakobi, 2018, 2019). Since cuing possible external and unstable attributions for ostracism may impact ostracism distress, death-anxiety could mediate this association.

### Overview of the Present Experiments

The three experiments presented here were designed to examine whether attributions of an ostracism experience to an external/internal stable/unstable cause could alleviate or perhaps eliminate ostracism distress in a factorial design based on Weiner’s (1972) causal dimensions of attribution. People who attribute negative events to internal, stable, and global causes (characterized as a maladaptive attribution style) are thought to be more susceptible to depressive reactions than people who attribute such events to contrasting causes (i.e., external, unstable; Mezulis et al., 2004). Empirical findings indicate that in achievement-related failure, a maladaptive attributional style was associated with depressive reactions (Metalsky et al., 1987) and with low aspirations and achievement (Peterson, 1990).

The decision to examine attribution effects in the reflective stage was motivated by Williams’ (2009) argument that immediate reactions to ostracism are resistant to moderation. By contrast, numerous studies have shown that people’s background and understanding of the context can enhance coping responses in the later reflective stage (e.g., Zadro et al., 2006; Wirth and Williams, 2009). Thus, it is reasonable to expect attribution to have a greater effect on alleviating distress in the reflective stage than immediately after the ostracism experience. This is because ostracized individuals are hypothesized to better implement attribution processes after they are given an opportunity to cognitively consider what prompted the ostracism and apply coping strategies, based on the robust notion that people need time to cognitively analyze new information (Zadro et al., 2006). Examining the four attributions served to identify the relative role of different attribution types in enhancing recovery from an ostracism episode. The findings may also constitute a foundation for the development of a useful intervention that enhances recovery after experiencing ostracism.

Experiment 1 examined whether exposure to one of the four types of attributions immediately after the ostracism event would moderate the effects of ostracism on victims’ mood. In Williams’ (2009) temporal need-threat theory of ostracism, ostracism also affects victims’ fundamental needs-satisfaction, and in particular their needs for a sense of belonging, self-esteem, control, and meaningful experience. Therefore, Experiment 2 examined the effects of victims’ exposure to the four types of attributions examined in Experiment 1 on needs-satisfaction. The use of two independent experiments and two study populations was aimed to enhance the generalizability and external validity of the results. The independent use of two well-known measures of ostracism distress was also designed to enhance the construct validity of the results and better eliminate biases such as the halo effect. This led to five hypotheses:

*Hypothesis 1:* Unstable attributions should reduce distress to a greater extent than stable attributions and when no explanation for ostracism is provided.

*Hypothesis 2:* External attributions should reduce distress to a greater extent than internal attributions and when no explanation for ostracism is provided.

*Hypothesis 3:* Participants provided with an external and unstable attribution should experience less distress when ostracized than when no attribution is provided. Conversely, when participants are given an internal and stable attribution, they should experience more distress than in all the other attributions, as well as the ostracism with no-attribution condition, and being included.

*Hypothesis 4a-b:* a. External-stable and b. internal-unstable attributions should reduce distress compared to internal attributions and when no explanation for ostracism is provided.

*Hypothesis 5:* The accessibility of death-related thoughts will mediate the relationship between attribution cue for the ostracism experience and distress.

The data in all experiments were included and no outliers were found. Assumptions regarding normality and homogeneity of variance were met in all the statistical analyses.

## Experiment 1

Experiment 1 was conducted to examine whether the type of attribution provided immediately after the ostracism experience (in the reflective stage) would moderate ostracism distress and whether certain types of attribution lead to complete recovery.

### Method

#### Participants and Design

An *a priori* power analysis to estimate the sample size was conducted (using G*Power 3.1; Faul et al., 2009). With an *α* = 0.05 and power = 0.80%, the projected sample size needed to detect a moderate–high effect size (*f* = 0.30) was approximately *N* = 149 for a between-group comparison (ANOVA). The actual sample size was larger than the calculated number of *N* = 149. Sample size was determined before any data analysis. All the participants who participated in the experiment were included in the analyses.

One hundred and ninety undergraduate business administration students (32% men; 90% unmarried), ranging in age from 19 to 42 (mdn = 24) volunteered to take part in the study. All the participants were recruited from an Israeli academic institution. No monetary compensation was provided. Participants were randomly assigned to one of six groups: four study groups based on a 2 (locus: internal, external) × 2 (stability: stable, unstable) between-subject design, and two control groups (an inclusion group and an ostracism with no-attribution group).

#### Materials and Procedure

The procedure was based on the multiple studies using the Cyberball game (e.g., Williams et al., 2000; Williams and Jarvis, 2006).

##### Cyberball Experience Manipulation

Participants were seated at a computer in separate cubicles. All instructions were presented on the screen. Participants were told that they were participating in a study about the relationships between mental visualization and task performance, and that these would be tested by means of a three-player internet ball-toss game called Cyberball. In this game, players engage in an animated ball-toss game. Depicted on the screen are two other ostensible players (represented by Cyber icons). The participant is represented as an animated hand at the bottom of the screen. Here, the participants were asked to use this game to engage in mental visualization. (They were encouraged to visualize the other players’ appearance and identity, the location of the game, and so on.) In total, there were 30 throws in each game. The Cyberball experience was manipulated by the number of ball tosses to the participant. In the five ostracism conditions, the participant received two tosses at the beginning of the game and then never received another toss. In the inclusion condition, the participant received one-third of the tosses (i.e., all players received an equal number of tosses).

Immediately after the participants in the four attribution groups completed the game, they were cued with one of the four types of attribution by a research assistant. For participants in the external-unstable attribution condition, the research assistant said, “Hey, I saw you did not get the ball very often, right?,” and after the participant concurred, she said to the participants “you did not have any luck today, did you?” For participants in the external-stable attribution condition, the research assistant said, “Hey, I saw you did not get the ball very often, right?,” and after the participant concurred, she stated, “This often happens on tasks like ball-toss games.” For participants in the internal-stable attribution condition, the research assistant said, “Hey, I saw you did not get the ball very often, right?,” and after the participant concurred, she stated, “This often happens depending on players’ personality.” For participants in the internal-unstable attribution condition, the research assistant said, “Hey, I saw you did not get the ball very often, right?,” and after the participant concurred, she stated that this might have happened because “I saw that you were not making much of an effort during the game.” Prior to the main experiments, 30 undergraduate students took part in a pilot study to test several cover stories for the four attribution types used in the current experiments. Twelve scenarios were presented (3 for each attribution type). Participants were asked to rate each scenario on two scales (stable–unstable and internal–external) from 1 to 5, which were the same scales used in the manipulation in the experiments themselves. The scenarios with the best statistical fit to each attribution type were selected for use in the main experiments^1^. To overcome order effects, the scenarios presented to participants were counterbalanced. In addition to this pilot, a manipulation check after the experiment was conducted which fully validated that the cover stories used in the two experiments corresponded to each attribution construct.

Two control groups were used: 1) An inclusion condition was used to examine whether attribution could lead to complete recovery and 2) an ostracism condition with no explanation was used to assess whether attribution would lead to greater distress than when an explanation was provided (e.g., internal-stable). In order to make all conditions similar except for the attribution manipulation, in the “ostracism with no explanation condition” the research assistant was instructed to say: “Hey, I saw you did not get the ball very often, right?,” and after the participant concurred, instead of providing one of the four possible causes, she asked participants to wait in another room as in all the other conditions. In the inclusion condition, the research assistant was instructed to say “Hey, I saw you got the ball often, right?,” and after the participant concurred, she also asked these participants to wait in a separate room. This procedure was used to minimize possible alternative explanations for the results other than the attribution manipulation. During this time, the research assistant ostensibly went to retrieve the questionnaire sheets and returned 10 min later. Participants were asked to wait a few minutes for the research assistant to come back, and not use their phones.

##### Dependent Variables

Participants completed anonymous self-reports on their emotional state based on the van Beest and Williams’s (2006) mood index, which contains four items assessing negative emotions (e.g., sad, hurt) and four assessing positive emotions (e.g., happy, elated; *α* = 0.91; see Appendix A for the complete scale). A 5-point scale was used. Positive emotions were reverse-scored; thus, a higher score on these items implied more distress.

As a check for the Cyberball manipulation, participants were asked to recall the percentage of ball tosses that they received in the game (0–100). To assess feelings of being ignored, participants responded to one item on a scale from 1 (*not at all*) to 5 (*very much so*). Exclusion was measured by one item on a scale from 1 (*not at all*) to 5 (*very much so*). These three items have been used extensively by Williams et al. in studies on ostracism (e.g., van Beest and Williams, 2006; Yaakobi and Williams, 2016a,b) and were translated into Hebrew independently by two native speakers of English and then back-translated to English according to the customary procedure for translation verification (Brislin, 1970).

As a check for the attribution manipulation, and the extent to which participants perceived the cover stories as correctly corresponding to the attribution constructs they were intended for, after completing the mood questionnaire the participants were asked to assess the research assistant’s explanation as to why they had not received the ball on two scales measuring locus from 1 (*internal*) to 5 (*external*) and stability from 1 (*stable*) to 5 (*unstable*). At the top of the questionnaire, the scales were explained to participants in parentheses (e.g., “internal = assigns the cause of the observed behavior to the person’s internal characteristics whereas external attribution assigns the cause of behavior to external factors”; “stable = assigns the cause to factors that are likely to happen again over time whereas unstable means that the factors can change”).

##### Demographics

Participants were also asked to complete a brief socio-demographic sheet indicating their gender, age and marital status. Marital status was included based on research showing that couplehood can contribute to mitigating the experience of ostracism (Yaakobi, 2018) and that marital status moderates the mediation effect of death anxiety on ostracism distress.

At the conclusion of the experiment, the participants were fully debriefed and were informed that they had played against preprogrammed computer players.

### Results and Discussion

#### Manipulation Checks

##### Cyberball Manipulation Check

To examine the Cyberball manipulation, a multivariate analysis of variance (MANOVA) for the dependent variables (percent throws received, feeling ignored/excluded) was conducted. As expected, the analysis yielded a significant effect for the Cyberball experience [Wilks’ lambda *F*(15, 489) = 10.82, *p* < 0.001, *ηp*^2^ = 0.232]. Ostracized participants reported that they received the ball on a smaller percentage of the tosses (*M* = 11.60%, *SD* = 13.12%) than the included participants (*M* = 34.40%, *SD* = 12.15%), *F*(1, 183) = 116.40, *p* < 0.001, *ηp*^2^ = 0.389; they also felt more ignored (*M* = 3.70, *SD* = 1.20) than the included participants (*M* = 1.98, *SD* = 1.23), *F*(1, 183) = 73.88, *p* < 0.001, *ηp*^2^ = 0.288) and more excluded (*M* = 3.67, *SD* = 1.25) than the included participants (*M* = 1.80, *SD* = 1.23), *F*(1, 183) = 83.15, *p* < 0.001, η*p*^2^ = 0.312. These findings confirmed that the Cyberball manipulation was successful.

##### Attribution Manipulation Check

To examine the attribution manipulation, a multivariate analysis of variance (MANOVA) for the dependent variables (classification of perceived cause of being ostracized: internal/external; stable/unstable) was conducted. As expected, the analysis yielded a significant effect for the attribution manipulation [Wilks’ lambda for locus *F*(3, 109) = 74.65, *p* < 0.001, *ηp*^2^ = 0.681; Wilks’ lambda for stability *F*(3, 109) = 79.12, *p* < 0.001, *ηp*^2^ = 0.693]. A Bonferroni *post hoc* analysis confirmed that the attribution manipulation was successful: Each group was significantly different (all *p*s < 0.001) from the other two groups having the opposite dimension, but were not significantly different from the other groups on the same dimension (all *p*s > 0.1; e.g., the internal-stable group reported effects that were significantly different from either internal-unstable or external-unstable groups on the stability dimension, but showed no significant difference from the internal-unstable group). Participants who were cued with an internal-stable cause for being ostracized evaluated the cause as more internal (*M* = 1.67, *SD* = 0.78) and stable (*M* = 2.00, *SD* = 0.85). Participants who were cued with an internal-unstable cause for being ostracized evaluated the cause as more internal (*M* = 1.96, *SD* = 0.77) and unstable (*M* = 4.13, *SD* = 0.76). Participants who were cued with an external-stable cause for being ostracized evaluated the cause as more external (*M* = 4.11, *SD* = 0.74) and stable (*M* = 1.96, *SD* = 0.74). Participants who were cued with an external-unstable cause for being ostracized evaluated the cause as more external (*M* = 4.11, *SD* = 0.71) and unstable (*M* = 4.26, *SD* = 0.68). (For full statistics on the Bonferroni *post-hoc* test, see Table 1.) As shown in Table 1, the results indicated that each condition successfully manipulated the dimension of interest.

**Table 1:** Values of *p* and 95% confidence interval values for the analysis of the manipulation check indices (internality–externality; stable–unstable) as a function of attribution manipulation (Experiment 1).

Dependent variable			*p*	95% LCI	95% UCI
Manipulation check Internal–external	internal-stable	internal-unstable	N. S.	−0.994	0.414
		external-stable	< 0.001	−3.123	−1.758
		external-unstable	< 0.001	−3.083	−1.801
	internal-unstable	internal-stable	N. S.	−0.414	0.994
		external-stable	< 0.001	−2.707	−1.594
		external-unstable	< 0.001	−2.657	−1.647
	external-stable	internal-stable	< 0.001	1.758	3.123
		internal-unstable	< 0.001	1.594	2.707
		external-unstable	N. S.	−0.475	0.472
	external-unstable	internal-stable	< 0.001	1.801	3.083
		internal-unstable	< 0.001	1.647	2.657
		external-stable	N. S.	−0.472	0.475
Manipulation check Stable–unstable	internal-stable	internal-unstable	< 0.001	−2.833	−1.428
		external-stable	N. S.	−0.645	0.716
		external-unstable	< 0.001	−2.900	−1.622
	internal-unstable	internal-stable	< 0.001	1.428	2.833
		external-stable	< 0.001	1.611	2.721
		external-unstable	N. S.	−0.634	0.373
	external-stable	internal-stable	N. S.	−0.716	0.645
		internal-unstable	< 0.001	−2.721	−1.611
		external-unstable	< 0.001	−2.769	−1.824
	external-unstable	internal-stable	< 0.001	1.622	2.900
		internal-unstable	N. S.	−0.373	0.634
		external-stable	< 0.001	1.824	2.769

*Time = N. S. – nonsignificant (p > 0.1)*.

Additional analyses were conducted to check the attribution manipulation. To determine whether locus was successfully manipulated, a 2 (Locus: internal vs. external) × 2 (Stability: stable vs. unstable) was conducted. The results indicated that only locus was significant [*F*(1, 105) = 203.34, *p* < 0.001; *ηp*^2^ = 0.668; *Minternal* = 1.82, *SD* = 0.77; *Mexternal* = 4.10, *SD* = 0.72], but not stability [*F*(1, 105) = 0.91, *p* = 0.343; *ηp*^2^ = 0.009; *Mstable* = 3.37, *SD* = 1.34; *Munstable* = 3.39, *SD* = 1.27] or the interaction between locus and stability [*F*(1, 105) = 0.53, *p* = 0.471; *ηp*^2^ = 0.005; Minternal*stable = 1.64, *SD* = 0.81; Minternal*unstable = 1.91, *SD* = 0.75; Mexternal*stable = 4.07, *SD* = 0.73; Mexternal*unstable = 4.11, *SD* = 0.71]. To determine whether stability was successfully manipulated, a 2 (Locus: internal vs. external) × 2 (Stability: stable vs. unstable) was conducted. The results indicated that only stability was significant [*F*(1, 105) = 190.72, *p* < 0.001; *ηp*^2^ = 0.654] (*Mstable* = 1.97, *SD* = 0.75; *Munstable* = 4.19, *SD* = 0.70), but not locus [*F*(1, 105) = 0.58, *p* = 0.447; *ηp*^2^ = 0.006; *Minternal* = 3.36, *SD* = 1.30; *Mexternal* = 3.40, *SD* = 1.30] or the interaction between stability and locus [*F*(1, 105) = 0.038, *p* = 0.845; *ηp*^2^ < 0.001; Minternal*stable = 1.91, *SD* = 0.83; Minternal*unstable = 4.09, *SD* = 0.75; Mexternal*stable = 2.00, *SD* = 0.73; Mexternal*unstable = 4.24, *SD* = 0.68]. Thus, the attribution manipulation was successful.

##### Mood

The means and standard deviations for the mood index are presented in Table 2.

**Table 2:** Means and Standard Deviations for the distress (mood) index as a function of the Cyberball experience for each of the four types of attribution and controls (Experiment 1).

	Ostracized (no explanation)			Locus			Included
				Internal		External		Total			
		M	SD	M	SD	M	SD	M	SD	M	SD
Controls		3.08	0.68							2.10	0.96
Stability	Unstable			2.53	0.91	2.47	0.92	2.49	0.91		
	Stable			3.70	0.98	2.35	0.74	2.75	1.02		
	Total			2.93	1.08	2.42	0.86	2.59	0.96		

*A higher score indicates higher distress. The sample sizes were as follows: (Internal-stable = 32 participants, internal-unstable = 31 participants, external-stable = 31 participants, external-unstable 32 participants, ostracized no explanation = 31 participants, included = 33). The participants were randomly assigned to the experimental and control groups; and no significant differences were found for age of gender between groups*.

**Figure 1:**
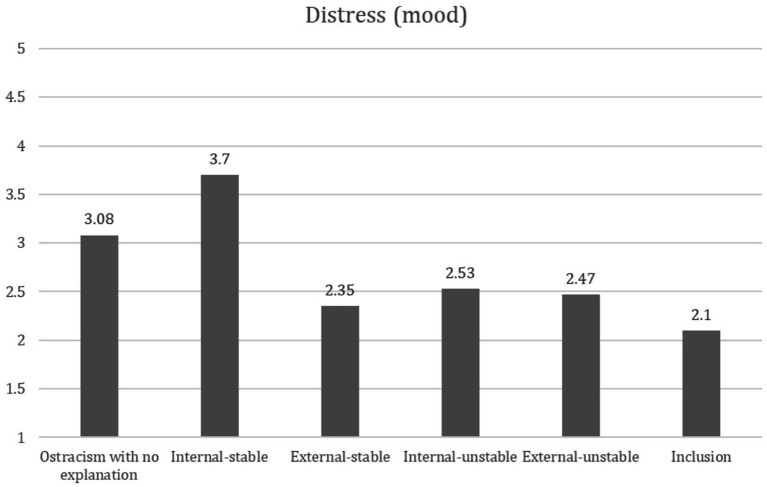
Mode as a function of attribution manipulation.

To examine whether the cued attribution type moderated ostracism distress, two analyses were conducted on mood. A 2 (locus: internal, external) × 2 (stability: stable, unstable) analysis of variance was conducted on mood. A one-way ANOVA for the six groups (internal-stable, internal-unstable, external-stable, external-unstable, included, no explanation) was conducted. The use of the 2 × 2 ANOVA served to capture both the main and interaction effects of the attribution manipulation. The one-way ANOVA captured the attribution effects in comparison with the inclusion and ostracism with no explanation control groups. The two-way ANOVA revealed a significant main effect for the locus manipulation on mood [*F*(1, 105) = 13.88, *p* > 0.001, *ηp*^2^ = 0.117] and a significant effect for the stability manipulation on mood [*F*(1, 105) = 7.58, *p* = 0.007, *ηp*^2^ = 0.067]. In addition, a significant interaction effect was found for the locus × stability manipulation [*F*(1, 105) = 11.51, *p =* 0.001, *ηp*^2^ = 0.099] on the mood measure. The one-way ANOVA also revealed a significant effect *F*(1, 184) = 9.13, *p < 0*.*001*. For a graphic presentation of the results, see Figure 1.

##### Ostracism With No Explanation vs. Inclusion

As expected, ostracized participants who were given no explanation for being ostracized showed a higher level of distress than the included participants (*p* < 0.001).

##### Stable vs. Unstable Attribution

A Bonferroni *post hoc* test revealed that ostracized participants who were cued with unstable attributions reported significantly less distress than participants who were cued with stable attributions (*p* = 0.038) and significantly less distress than participants who were given no explanation for being ostracized (*p* = 0.030). The mood of participants who were cued with unstable attributions was similar to the mood of the included participants (*p* = 0.097), which is comparable to complete recovery. Participants who were cued with stable attributions reported a similar level of distress as ostracized participants who were given no explanation for being ostracized (*p* > 0.10), but reported a significantly higher level of distress than that of included participants (*p* < 0.001).

Thus overall, *an unstable attribution not only alleviated ostracism distress, which fully supported H1, but led to complete recovery*. *A stable attribution led to a similar level of perceived distress as for participants who did not receive any explanation for the cause of ostracism*.

##### Internal vs. External Attribution

A Bonferroni *post hoc* test revealed that ostracized participants who were cued with external attributions reported significantly less distress than participants who were cued with internal attributions (*p* = 0.002) and significantly less distress than participants who were given no explanation for being ostracized (*p* = 0.006). Participants in the external attribution conditions reported a mood level similar to the included participants (*p* = 0.338), which is comparable to complete recovery. Participants who were cued with internal attributions reported a level of distress comparable to that of ostracized participants who were given no explanation for being ostracized (*p* > 0.10), but reported a significantly higher level of distress than included participants (*p* < 0.001).

Thus overall, *external attributions not only alleviated ostracism distress, fully supporting H2, but led to complete recovery when measured shortly after the cued attribution of ostracism*. *Internal attributions led to a similar level of perceived distress as when not receiving any explanation for the cause of ostracism*.

##### Locus × Stability Attribution

A Bonferroni *post hoc* analysis of the interaction between locus × stability revealed that participants who thought they did not receive the ball for external-unstable reasons reported lower distress than ostracized participants who thought they did not receive the ball for internal-stable reasons (*p* < 0.001) and marginally lower distress than participants who received no explanation (*p* = 0.073), but their reported level of distress was not significantly different from the distress reported by the included participants (*p* = 0.586).

Moreover, participants who thought they did not receive the ball for external-stable reasons reported lower distress than ostracized participants who received no explanation for being ostracized (*p* = 0.037) and lower distress than participants who thought they had not received the ball for internal-stable reasons (*p* < 0.001); their reported level of distress was not significantly different from the distress reported by included participants (*p* > 0.10).

In addition, participants who thought they did not receive the ball for internal-stable reasons reported similar distress as ostracized participants who received no explanation for being ostracized (*p* = 0.867), but significantly higher distress than the included participants (*p* < 0.001) and participants in all the other attribution groups (all *p*’s < 0.005), thus supporting the second part of H3 regarding all other attributions cued, and reported similar (but not lower distress) than ostracized participants who received no explanation for being ostracized.

In addition, participants who thought they did not receive the ball for internal-unstable reasons reported lower distress than ostracized participants who thought they did not receive the ball for internal-stable reasons (*p* = 0.005), but did not differ in their level of distress from participants who received no explanation for being ostracized (*p* > 0.10). Thus, although not hypothesized, an internal-unstable attribution did not lead to less distress than when participants were not provided with an explanation for being ostracized. They, however, reported less distress than participants cued to the internal-stable attribution.

Thus, *external-stable attributions decreased ostracism distress and led to complete recovery*. *External-unstable attributions led to same level of distress as included participants*. Thus, H3 was partially supported, and H4a was fully supported, and revealed that although not hypothesized, external-stable attributions eliminated distress altogether. H4b was partially supported.^2^

Overall, these findings confirmed that causal attributions for ostracism provided immediately after the ostracism episode moderated the effects of ostracism distress on a mood questionnaire administered soon after the experience. Specifically, the results indicated that *cuing external, unstable, external-stable, or external-unstable causes as explanations for ostracism led to similar mood levels as when included*. *Providing external or unstable causes for ostracism led to less distress than providing internal causes or not providing any cues as to the cause of ostracism*. As hypothesized, *internal-stable attributions led to higher distress than all the other* attributions, but these participants did not differ from ostracized participants who received no explanation for being ostracized. Finally, the findings also point to the differential role of attribute types in alleviating distress after an ostracism episode. However, as Williams (2009) reported in numerous studies (e.g., see meta-analyses of Hartgerink et al., 2015), ostracism also threatens fundamental needs. Experiment 2 was conducted to determine whether cuing different attributions would yield similar results for needs-satisfaction as for mood.

## Experiment 2

Experiment 2 was conducted to increase the generalizability of the results of Experiment 1 with respect to external and construct validity. Experiment 2 examined whether the different types of attribution cued as possible explanations for ostracism would also affect participants’ fundamental needs-satisfaction (Williams, 2009) in the post-ostracism reflective stage. Similar to the procedure in Experiment 1, Experiment 2 examined whether specific attributions would lead to greater needs-satisfaction than when no explanation for the cause of ostracism was given, or would lead to a level of needs-satisfaction similar to the needs-satisfaction of included participants.

### Method

#### Participants and Design

To determine the sample size for Experiment 2, the same procedure as in Experiment 1 was used. The actual sample size was larger than the calculated number of N = 149. Sample size was determined before any data analysis. As in Experiment 1, all the participants were included in the analyses. One hundred and ninety-four undergraduate business administration students (40% men, 70% unmarried), ranging in age from 20 to 43 (mdn = 25) volunteered to take part in the study. All participants were recruited from an Israeli academic institution. No monetary compensation was provided. Participants were randomly assigned to six groups: four groups in a 2 (locus: internal, external) × 2 (stability: stable, unstable) between-subject design, and two control groups (an inclusion group and a no-attribution group). Three participants whose stated attribution did not correspond to their assigned group on the attribution check were dropped from the analyses.

#### Materials and Procedure

The general outline for the procedure, the experimental manipulation, and the measures were identical to those used in Experiment 1. The only difference was that fundamental needs-satisfaction was used as the dependent variable instead of the mood measure. After the research assistant returned to the room where the participants were waiting, the participants provided anonymous self-reports on their current levels of satisfaction of their needs for belonging (e.g., “I felt I belonged to the group” (reversed); I felt rejected), self-esteem (e.g., “I felt good about myself” (reversed) “I felt liked” (reversed)), meaningful existence (e.g., “I felt important” (reversed), “I felt invisible,” and control) (e.g., “I felt powerful” (reversed), “I felt I had control over the course of the game”(reversed)), on the 5-point Need Satisfaction Scale developed by van Beest and Williams (2006) (*α* = 0.95; see Appendix B for the complete scale). Finally, we confirmed that participants had understood the key elements of the experiment.

### Results and Discussion

#### Manipulation Checks

##### Cyberball Manipulation Check

To examine the Cyberball manipulation, a multivariate analysis of variance (MANOVA) for the dependent variables (percent throws received, feeling ignored/excluded) was conducted. As expected, the analysis yielded a significant effect for the Cyberball experience [Wilks’ lambda *F*(15, 486) = 74.65, *p* < 0.001, *ηp*^2^ = 0.262]. Ostracized participants reported that they received the ball on a smaller percentage of tosses (*M* = 9.60%, *SD* = 10.65%) than the included participants (*M* = 34.30%, *SD* = 12.53%), *F*(1, 182) = 180.32, *p* < 0.001, *ηp*^2^ = 0.498; they also felt more ignored (*M* = 3.70, *SD* = 1.16) than the included participants (*M* = 1.92, *SD* = 1.20), *F*(1, 182) = 84.93, *p* < 0.001, *ηp*^2^ = 0.318, and felt more excluded (*M* = 3.66, *SD* = 1.18) than the included participants (*M* = 1.81, *SD* = 1.19). Thus, the Cyberball manipulation was successful.

##### Attribution Manipulation Check

To examine the attribution manipulation, a multivariate analysis of variance (MANOVA) for the dependent variables (attribution type: internal/external; stable/unstable) was performed. As in Experiment 1, this analysis served to examine both factors simultaneously to ensure that only the factor of interest was affected. As expected, the analysis yielded a significant effect for the attribution manipulation [Wilks’ lambda for locus *F*(3, 91) = 43.72, *p* < 0.001, *ηp*^2^ = 0.601; Wilkes’ lambda for stability *F*(3, 91) = 50.04, *p* < 0.001, *ηp*^2^ = 0.633]. A Bonferroni *post hoc* analysis confirmed that the attribution manipulation was successful since each group was significantly different (all *p*’s < 0.001) from the other two groups representing the opposite dimension, but was not significantly different from the other group on the same dimension (all *p*’s > 0.1; e.g., internal-stable was significantly different from both internal-unstable and external-unstable on the stability dimension, but was not significantly different from the internal-unstable group on the locus dimension). Participants who were cued with an internal-stable cause for being ostracized evaluated the cause as more internal (*M* = 2.10, *SD* = 0.99) and stable (*M* = 1.50, *SD* = 0.71). Participants who were cued with an internal-unstable cause for being ostracized evaluated the cause as more internal (*M* = 1.74, *SD* = 0.56) and unstable (*M* = 4.05, *SD* = 0.85). Participants who were cued with an external-stable cause for being ostracized evaluated the cause as more external (*M* = 4.04, *SD* = 1.02) and stable (*M* = 1.72, *SD* = 0.74). Participants who were cued with an external-unstable cause for being ostracized evaluated the cause as more external (*M* = 3.97, *SD* = 0.76) and unstable (*M* = 4.10, *SD* = 1.10; for full statistics of the Bonferroni *post hoc* test see Table 3). As shown in Table 3, each condition was successfully manipulated on the dimension of interest.

**Table 3:** Values of *p* and 95% confidence interval values for the analysis of the manipulation check indices (internality–externality; stable–unstable) as a function of attribution manipulation (Experiment 2).

Dependent variable			*p*	95% LCI	95% UCI
Manipulation check Internal–external	internal-stable	internal-unstable	N. S.	−0.517	1.243
		external-stable	<0.001	−2.783	−1.097
		external-unstable	<0.001	−2.676	−1.070
	internal-unstable	internal-stable	N. S.	−1.243	0.517
		external-stable	<0.001	−2.989	−1.618
		external-unstable	<0.001	−2.872	−1.601
	external-stable	internal-stable	<0.001	1.097	2.783
		internal-unstable	<0.001	1.618	2.989
		external-unstable	N. S.	−0.516	0.650
	external-unstable	internal-stable	<0.001	1.070	2.676
		internal-unstable	<0.001	1.601	2.872
		external-stable	N. S.	−0.650	0.516
Manipulation check Stable–unstable	internal-stable	internal-unstable	<0.001	−3.526	−1.579
		external-stable	N. S.	−1.152	0.712
		external-unstable	<0.001	−3.496	−1.720
	internal-unstable	internal-stable	<0.001	1.579	3.526
		external-stable	<0.001	1.574	3.091
		external-unstable	N. S.	−0.759	0.648
	external-stable	internal-stable	N. S.	−0.712	1.152
		internal-unstable	<0.001	−3.091	−1.574
		external-unstable	<0.001	−3.033	−1.743
	external-unstable	internal-stable	<0.001	1.720	3.496
		internal-unstable	N. S.	−0.648	0.759
		external-stable	<0.001	1.743	3.033

*Time = N. S. – nonsignificant (p > 0.1)*.

As in Experiment 1, additional analyses were conducted to check the attribution manipulation. To determine whether locus was successfully manipulated, a 2 (Locus: internal vs. external) × 2 (Stability: stable vs. unstable) ANOVA was conducted to evaluate locus. The results indicated that only locus was significant [*F*(1, 106) = 96.13, *p* < 0.001; *ηp*^2^ = 0.488; *Minternal* = 1.86, *SD* = 0.76; *Mexternal* = 4.00, *SD* = 0.87], but not stability [F(1, 106) = 1.10, *p* = 0.297; *ηp*^2^ = 0.011] (*Mstable* = 3.50, *SD* = 1.34; *Munstable* = 3.20, *SD* = 1.28) or the interaction between locus and stability [F(1, 106) = 0.51, *p* = 0.478; *ηp*^2^ = 0.005] (Minternal*stable = 2.11, *SD* = 1.05; Minternal*unstable = 1.74, *SD* = 0.56; Mexternal*stable = 4.04, *SD* = 1.02; Mexternal*unstable = 3.97, *SD* = 0.77). Thus, only the manipulation of locus was significant.

To determine whether stability was successfully manipulated, a 2 (Locus: internal vs. external) × 2 (Stability: stable vs. unstable) ANOVA was conducted to evaluate stability. The results revealed that only stability was significant [*F*(1, 105) = 122.20, *p* < 0.001; *ηp*^2^ = 0.533; *Mstable* = 1.63, *SD* = 0.71; *Munstable* = 4.08, *SD* = 0.99], but not locus [F(1, 105) = 0.95, *p* = 0.331; *ηp*^2^ = 0.009] (*Minternal* = 3.18, *SD* = 1.49; *Mexternal* = 3.26, *SD* = 1.48) or the interaction between stability and locus [*F*(1, 105) = 0.63, *p* = 0.431; *ηp*^2^ = 0.006; Minternal*stable = 1.33, *SD* = 0.50; Minternal*unstable = 4.05, *SD* = 0.85; Mexternal*stable = 1.74, *SD* = 0.75; Mexternal*unstable = 4.10, *SD* = 1.06]. Thus, only the manipulation of stability was significant. Overall, the results revealed that the attribution manipulation was successful.

##### Needs-Satisfaction

The means and standard deviations for the needs-satisfaction index are presented in Table 4.

**Table 4:** Means and Standard Deviations for the distress (needs satisfaction) index as a function of the Cyberball experience for each of the four types of attribution and controls (Experiment 2).

Ostracized (no explanation)		Locus						Included	
				Internal		External		Total			
		M	SD	M	SD	M	SD	M	SD	M	SD
Controls		3.34	0.61							2.41	0.89
Stability	Unstable			2.78	0.68	2.71	0.89	2.74	0.82		
	Stable			4.12	0.54	2.84	0.64	3.22	0.85		
	Total			3.26	0.90	2.76	0.80	2.84	0.89		

*A higher score indicates higher distress. The sample sizes were as follows (Internal-stable = 33 participants, internal-unstable = 31 participants, external-stable = 32 participants, external-unstable 32 participants, ostracized no explanation = 34 participants, included = 33). The participants were randomly assigned to the experimental and control groups; and no significant differences were found for age of gender between groups*.

**Figure 2:**
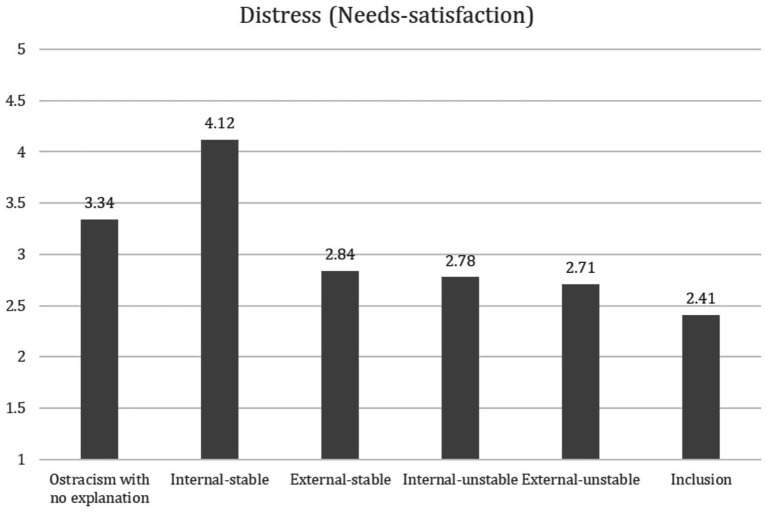
Needs satisfaction as a function of attribution manipulation.

To examine whether the attributed cause of the ostracism episode moderated distress, two analyses were performed on needs satisfaction. A 2 (locus: internal, external) × 2 (stability: stable, unstable) analysis of variance was conducted on needs-satisfaction for attributions and an additional one-way ANOVA was performed for the six study conditions (internal-stable, internal-unstable, external-stable, external-unstable, included, and no-attribution). The two-way ANOVA revealed a significant main effect on needs-satisfaction for the locus manipulation [*F*(1, 102) = 8.95, *p* > 0.001, *ηp*^2^ = 0.079] and a significant effect for the stability manipulation [*F*(1, 102) = 17.64, *p* < 0.001, *ηp*^2^ = 0.092]. In addition, a significant interaction effect on the needs-satisfaction measure was found for the locus × stability manipulation [*F*(1, 102) = 12.01, *p =* 0.001, *ηp*^2^ = 0.065]. The one-way ANOVA also revealed a significant effect *F*(1, 179) = 11.88, *p < 0*.001. For a graphic presentation of the results, see Figure 2.

##### Stable vs. Unstable Attribution

A Bonferroni *post hoc* test revealed that the ostracized participants cued with unstable causes reported significantly lower distress than participants who were cued with stable causes (*p* = 0.017) and participants who were provided no explanation for being ostracized (*p* = 0.004). Moreover, participants who were cued with unstable causes showed similar distress as participants in the included condition (*p* = 0.156), thus presenting complete recovery for needs-satisfaction as was the case for mood in Experiment 1. However, participants cued with stable causes reported a similar level of distress as ostracized participants who were given no explanation (*p* > 0.10), but a significantly higher level of distress than included participants (*p* < 0.001).

*Thus, similar to Experiment 1, H1 was fully supported, revealing that unstable attributions proved to be a sufficient intervention to alleviate distress*. *Unstable attribution not only alleviated ostracism distress, but led to complete recovery after a short delay as in Experiment 1*. *A stable attribution led to a similar level of perceived distress as when not receiving any explanation for the cause of ostracism*.

##### Internal vs. External Attribution

A Bonferroni *post hoc* test revealed that ostracized participants who were provided with an external attribution reported significantly less distress (greater needs-satisfaction) than participants whose ostracism was attributed to an internal cause (*p* = 0.022) and participants who were given no explanation (*p* = 0.006). Moreover, as in Experiment 1, participants who were cued with an external attribution showed similar distress levels as included participants on the needs-satisfaction scale (*p* = 0.084), and thus evidenced complete recovery. As in Experiment 1, participants who were cued with internal causes reported similar distress as the ostracized participants who were given no explanation (*p* > 0.10), but significantly higher distress than the included participants (*p* < 0.001). As expected, ostracized participants who were given no explanation reported higher distress than included participants (*p* < 0.001).

*External attributions alleviated ostracism distress, thus fully supporting H2 and also led to complete recovery for needs-satisfaction, as was the case for mood*. *Internal attribution led to similar levels of perceived distress as when not receiving any explanation for the cause of ostracism, as found in Experiment 1*.

##### Locus × Stability Attribution

A Bonferroni *post hoc* analysis of the interaction between locus and stability revealed that participants cued with an external-unstable cause reported less distress than ostracized participants who received no explanation (*p* = 0.016) or those cued to an internal-stable attribution (*p* < 0.001) and their reported level of distress did not differ significantly from included participants (*p* > 0.10).

Thus, cuing an internal-unstable attribution reduced ostracism distress, supporting the first part of H3.

Participants cued with an external-stable cause reported lower distress than ostracized participants cued to an internal-stable cause (*p* < 0.001) and reported similar distress as included participants (*p* > 0.10), but surprisingly did not differ from the ostracized participants who received no explanation (*p* = 0.283). Thus, H4a was partially supported. Their reported distress was similar to the ostracized participants cued to an external-unstable cause (*p* > 0.10).

In addition, participants cued to an internal-unstable cause reported similar distress as ostracized participants who received no explanation (*p* = 0.218) and did not differ in their distress from the included participants (*p* > 0.10). Thus, the internal-unstable attribution did not reduce ostracism distress, contrary to what was predicted in H4b.

Finally, participants cued with an internal-stable cause reported higher distress than ostracized participants cued to an internal-unstable cause (*p* < 0.001), but did not differ from ostracized participants who received no explanation (*p* = 0.073), but reported higher distress than included participants (*p* < 0.001). They also reported higher distress than ostracized participants cued to an internal-unstable cause (*p* < 0.001), ostracized participants cued to an external-stable cause (*p* < 0.001), and ostracized participants cued to an external-unstable cause (*p* < 0.001).

Thus, internal-stable attribution led to similar distress as when not receiving any explanation for the cause of ostracism and higher distress than all other types of attribution.

Overall, these findings confirmed that attributions moderated the effects of ostracism on needs-satisfaction. Cuing unstable, external, and external-unstable causes of ostracism reduced ostracism distress and led to complete recovery with respect to needs-satisfaction reported after a short delay, thus supporting H1, H2, and H3. In addition, internal-stable attributions of ostracism led to the greatest distress, similar to when participants received no explanation for being ostracized. Finally, H4a regarding the moderating role of external-stable attribution effects was only supported for mood and partially supported for needs-satisfaction. H4b predicting the moderating role of internal-unstable attribution effects was not supported.

## Experiment 3

Experiment 3 was conducted to examine the possible mediating role of death-anxiety on the association between cuing a cause for attribution and ostracism distress.

### Method

#### Participants and Design

To determine the sample size for Experiment 3, the same procedure as in Experiments 1 and 2 was used. The actual sample size was larger than the calculated number of *N* = 149. Sample size was determined before any data analysis. Two hundred and seventy-two undergraduate business administration students (35% men), ranging in age from 19 to 51 (mdn = 24) volunteered to take part in the study. All participants were recruited from an Israeli academic institution. No monetary compensation was provided. Participants were randomly assigned to six groups: four groups in a 2 (locus: internal, external) × 2 (stability: stable, unstable) between-subject design, and two control groups (an inclusion group and a no-attribution group). After a delay and before completing the distress measures (needs satisfaction and mood), the accessibility of death-related thoughts scale was completed by participants.

#### Materials and Procedure

The general outline for the procedure, the experimental manipulation, and the measures were identical to those used in Experiments 1 and 2.

The accessibility of death-related thoughts was assessed by a Hebrew version of the word completion task originally devised in English by Greenberg et al. (1994) and successfully used in Hebrew by Mikulincer and Florian (2000) on an Israeli sample. In this study, the task consisted of 20 Hebrew word fragments that participants were asked to complete with the first word that came to mind by filling in one missing letter. Eight of the twenty Hebrew fragments could be completed to form either neutral or death-related Hebrew words. For example, participants saw the Hebrew fragment _VEL and could complete it with the Hebrew word HVEL (“cord”) or with the death-related EVEL (“mourning”). The possible death-related words were the Hebrew words for death, mourning, cadaver, grave, killing, dying, grief, and skeleton. The dependent measure was the number of death-related Hebrew words (0–8) completed by each participant.

### Results and Discussion

To examine the mediational role of death anxiety, the PROCESS macro (Hayes, 2013) Model 4 was used to calculate two sets of regressions. The first set of regressions examined the association between the predictors and mediator variables. The second set of regressions examined the links from the mediators to the outcomes. To test the significance of the indirect effects of attribution on ostracism distress through death-related thoughts, the bootstrapping approach was used and calculated the 95% CI for the indirect effects on 5,000 resamples (Hayes, 2013).

Table 5 presents the regression results for simple mediation of accessibility of death-related words on the association between attribution on either mood or needs-satisfaction (Experiment 3).

**Table 5:** Regression results for simple mediation of ADW on the association between attribution and mood (Experiment 3).

	Locus						Stability					
Variable	*β*	*SE*	*t*	*p*	*LLCI*	*ULCI*	*β*	*SE*	*t*	*p*	*LLCI*	*ULCI*
*Direct and total effects*												
Distress regressed on Attribution:	0.02	0.04	0.61	0.545	−0.052	0.099	0.02	0.06	0.36	0.720	−0.090	0.130
ADW regressed on attribution:	−0.12	0.06	−2.04	0.043	−0.230	−0.004	−0.01	0.09	−0.02	0.987	−0.169	0.166
Distress regressed on ADW, controlling for attribution:	−0.11	0.04	−2.76	0.006	−0.190	−0.032	−0.14	0.05	−2.59	0.011	−0.248	−0.033
Distress regressed on attribution, controlling for ADW:	0.02	0.04	0.61	0.545	−0.052	0.099	0.02	0.06	0.359	0.720	−0.090	0.130
		***β***	***SE***	***LLCI***	***ULCI***	***β***	***SE***			***LLCI***	***ULCI***	
*Indirect effects and significance using normal distribution*												
*Bootstrap results for indirect effects*												
Effect		*0.01*	*0.01*	*0.001*	*0.034*	*0.01*	*0.01*			*−0.027*	*0.025*	
Distress regressed on Attribution:	0.07	0.04	1.82	0.070	−0.006	0.144	0.08	0.05	1.52	0.131	−0.024	0.196
ADW regressed on attribution:	−0.10	0.06	−1.65	0.100	−0.207	0.018	0.02	0.08	0.21	0.836	−0.146	0.180
Distress regressed on ADW, controlling for attribution:	0.10	0.04	2.52	0.012	0.022	0.181	0.06	0.05	1.17	0.243	−0.043	−0.167
Distress regressed on attribution, controlling for ADW:	0.07	0.04	1.82	0.070	−0.006	0.144	0.08	0.05	1.52	0.131	−0.024	0.186
		***β***	***SE***	***LLCI***	***ULCI***	***β***	***SE***			***LLCI***	***ULCI***	
*Indirect effects and significance using normal distribution*												
*Bootstrap results for indirect effects*												
Effect		−0.01	0.01	−0.028	0.002	0.01	0.01			−0.013	0.016	

*Standardized regression coefficients are reported. Bootstrap sample size = 5,000. AWD – accessibility of death-related words. LL, lower limit; CI, confidence interval; UL, upper limit*.

External attribution was negatively associated with the accessibility of death-related thoughts, as indicated by the significant unstandardized regression coefficient. There was a negative relationship between accessibility of death-related thought and mood, when controlling for attribution. External attribution had an indirect effect on mood: this indirect effect was positive, as hypothesized. The formal two-tailed significance test (assuming a normal distribution) indicated that the indirect effect was significant. Bootstrap results showed that the bootstrapped 95% CI around the indirect effect did not include zero. Thus, Hypothesis 5 was fully supported for external attribution on mood through accessibility of death-related thoughts. In contrast, there was no mediation of death-related thoughts for external attribution on needs satisfaction or for unstable attribution on either mood or needs-satisfaction (see Table 5).

## General Discussion

These three experiments are the first to provide empirical evidence for an effective intervention to eliminate ostracism distress by cuing possible attributions for being ostracized in the reflective stage. The findings underscore the differential effect of different types of attribution described in Weiner’s influential taxonomy on mood and needs-satisfaction, two factors that are known to be related to ostracism distress. Specifically, attributing an ostracism episode to an unstable or external cause led to complete recovery from ostracism distress for mood and needs-satisfaction. Not surprisingly, on all measures, cuing ostracized participants with internal-stable attributions led to the highest distress. However, their level of distress was similar to participants who were not provided with a cause for being ostracized and not lower than when not given any explanation. Thus, people may experience psychological effects from being ostracized that lead to the same level of distress as when “blaming” their personality for being ostracized. Neurological findings suggest that the detection of ostracism activates the same region as physical pain (Eisenberger et al., 2003; Eisenberger, 2013). Hence, being ostracized could activate an immediate internal-state explanation. This is consistent with the fundamental attribution error of attributing behavior to internal causes (Ross, 1977).

The results also showed that a combination of locus and stability moderated distress as a function of the specific distress measure. Cuing with an external-unstable attribution was the most effective for needs-satisfaction, whereas external-stable cuing was the most effective for mood. Note that the research assistant talked as much to the control group participants who received no cues as to participants in the attribution conditions. This served to minimize possible alternative explanations for the results other than the attribution manipulation and avoided potential confounds for the effects. Finally, the results revealed that death anxiety mediated the effects of cuing external attribution for ostracism on mood reduction after ostracism.

The current experiments make theoretical and practical contributions. The results contribute to work on ostracism by identifying the ways in which attributions can moderate ostracism effects. These experiments respond to Williams’ (2009) call for “more research to determine the recovery rate as a function of the attributed ostracism motive” (p. 296), and other researchers’ similar recommendations (e.g., Park et al., 2017). The current findings enable a better understanding of the relative importance and effectiveness of four different attributions in alleviating and eliminating distress. They enhance both literature and practice by outlining possible principles for an intervention that can reduce the extensive negative effects of ostracism. They suggest that a targeted intervention based on a cognitive interpretation (e.g., through attribution processes) administered immediately after the ostracism episode can alleviate distress which for specific attributions results in complete recovery after a short delay. Numerous ostracism studies have pointed to the effects of situational and individual moderators in the reflective stage. This may imply that ostracism victims’ disposition, background, and grasp of context can orient their coping responses and impact the speed of their recovery (Zadro et al., 2006). The current findings are consistent with these claims but also reveal that attribution processes can reduce distress resulting from ostracism. These may point to new research directions related to these moderation effects and the mechanisms involved in mitigating the deleterious effects of ostracism.

Although attribution may be perceived as a stable personality variable, the findings here suggest that an external intervention can alter the accessibility of explanations for being ostracized by highlighting other possible attributions. Thus, these results also contribute to attribution theory in that attribution can be manipulated. However, future research should examine the effects of shifts in attribution at more distant time points from the ostracism experience. If recovery appears in the reflective stage, only a few minutes after the ostracism experience, it may lead to recovery in the long run. Future research should examine this empirically as well as whether the other attributions that were found to lower, but not completely eliminate levels of distress can nevertheless contribute to recovery in the longer run.

The results make practical contributions as well by shedding light on ways in which victims can be helped to recover from the negative consequences of ostracism. Providing an explanation that shifts the responsibility for the episode to an external factor or to temporary (unstable) circumstances may be one way to restore victims’ well-being. Psychologists, consultants, managers, parents, and teachers can draw on the mechanisms reported here to facilitate and accelerate recovery from ostracism. Future studies should extrapolate the findings to such practices as attributional retraining therapy (Hamm et al., 2014). As in CBT therapy, one of the treatment mechanisms consists of providing clients with tools such as viewing a (negative) experience from different perspectives. The current findings may suggest that the negative experience of ostracism can be mitigated by suggestions of different attributions. Therapists working with victims of ostracism could possibly suggest considering other possible causes for being ostracized, and external/unstable attributions in particular. Previous findings have indicated that individuals’ dominant attribution styles moderate the relationship between daily hassles and anxiety and depression symptoms (Wang, 2021). Note that hopelessness theory, one of the main cognitive models of depression describes the relationship between the onset and maintenance of depression and a dysfunctional and rigid attributional style (Abramson et al., 1989). Heggeness et al. (2020) argued that negative attributional biases can lead to the excessive misperception that stressors are insurmountable. They report greater negative outcomes when a controllable stressor is misconstrued as persistent (i.e., stable) and when the cause is attributed to the self (i.e., internal). Hence, more depressive individuals may have higher risk of interpreting ostracism as arising from internal-stable factors, thus making this group more vulnerable to victimhood.

The discounting principle (Kelly, 1972) refers to the cognitive process of reducing a belief in one potential cause of behavior (e.g., “I did not get the ball because others did not like me”) by substituting another viable cause (e.g., “There was an internet problem and therefore the other players may have not seen me”). Thus, providing specific types of attributions for an ostracism episode could augment victims’ well-being by rectifying possible self-blame. If there is an external and/or unstable cause for ostracism, it is important to highlight it.

To implement more systematic coping and recovery, victims of ostracism need both the motivation and the ability to do so (e.g., the ELM model, Petty and Cacioppo, 1986). Future studies could extend these ostracism results to pathologies and examine whether major depressive individuals who suffer from less motivation, for example, can muster the extra effort to recover from ostracism distress through changes in attribution. In cases of learned helplessness, attribution processes may be less effective. This should be examined in future research.

Finally, research has found that psychological flexibility moderates ostracism distress and that techniques to enhance individuals’ psychological flexibility can help them cope with ostracism (Waldeck et al., 2017). These authors reported that the relationship between perceived ostracism and distress only appeared when psychological flexibility was low but not high. Thus, people who are more prone to adopting methods that hamper psychological flexibility when coping with stressors may be more likely to experience distress. In contrast, high psychologically flexible individuals may cope better and recover more quickly from ostracism. Given the increasing interest in possible ostracism moderators, future research should also concentrate on developing a holistic model for coping with ostracism.

### Limitations and Future Work

The current experiments have limitations that deserve mention. One limitation relates to self-reported measures. Although the scales used here are extremely common and are the most widely used measures assessing the impact of ostracism in ostracism research (e.g., Garczynski and Brown, 2014; for meta-analyses see Hartgerink et al., 2015), participants may have underreported their negative mood and unsatisfied needs. For example, Garczynski and Brown (2014) found that temporal framing, or phrasing ostracism self-reported measures shaped individuals’ responses. They argued that “differences based on tense are the result of biased self-reports (due to social desirability concerns or implicit theories of change over time), rather than representing actual recovery from exclusion” (p. 40). They noted that greater distress will be reported when asking participants to report their feelings in the past than in the present. While it is possible that people may try to create an impression by falsely answering self-reports, the use of a between-subject design and a mental visualization cover story make it less likely that participants knew what sort of relative impression to make. They were unaware of the other conditions and could not know whether they were reporting scores that were more or less comparable to other conditions. Moreover, the current paper focuses solely on the reflective stage, during which the participant can appraise the experience of being ostracized, such as its cause (Williams, 2007). In the experiments, all the participants were asked to relate to their current feelings (see Appendices A, B). However, as also suggested by Garczynski and Brown (2014) future studies should assess distress using physiological measures such as levels of cortisol or blood pressure while participants report their feelings. To examine the tense effects, future research could also manipulate self-presentation beliefs and participants’ concerns about emotional intensity across time, or counterbalance the tenses of the measures (see details in Garczynski and Brown, 2014). Nonetheless, future research should use implicit or psychological measures to examine the effects of attribution on ostracism distress and also examine the mitigating effects of attribution on actual post-ostracism behavior. It would also be important to examine whether being ostracized by someone the participant knows exacerbates the distress of ostracism and decreases the effects of attribution found here. The examination should also be extended to include the more recent expansion of Weiner’s attribution theory to eight possible types of attribution.

Another limitation relates to the fact the current studies used an experimental design with a brief (but externally valid) manipulation of ostracism (see the meta-analysis of nearly 120 Cyberball studies by Hartgerink et al., 2015). As such, the generalizability of the findings can be challenged. However, the participants were heterogeneous in terms of their gender and age. Future research should test the moderating role of attribution on ostracism effects on a broader spectrum of populations while using other manipulations (or indices) of ostracism. It would be interesting to examine whether attribution interventions are also effective when ostracism occurs with people who are more emotionally close to individuals, such as their peers and romantic partners. Moreover, in the current research the intervention was administered immediately after the ostracism experience. Future research should also examine whether attributional interventions that are administered after a longer delay have similar or different effects than those found here.

In Weiner’s (1985) attribution theory, controllability was defined as another attribution factor. As reported by Warburton et al. (2006), ostracized individuals can become aggressive to establish control. Thus, the importance of a sense of control over ostracism affects recovery. In the current study, only locus and stability were analyzed.

Since previous findings have indicated that death anxiety mediates the association between ostracism experience and distress (e.g., Yaakobi, 2018, 2019), future research should examine whether death anxiety moderates attribution effects using an experimental design.

Finally, it would be of value to examine whether the moderating effects found in the current study in the reflective stage are sustained in what Williams (2009) calls the resignation stage, and if not, when this moderating effect dissipates. According to Williams’ (2009) temporal need-threat model, the third (resignation) stage only characterizes individuals who are chronically exposed to ostracism. Their resources are depleted, which leads to internalized feelings of alienation, depression, helplessness, and worthlessness. If the effects of attribution help mitigate ostracism distress immediately after the experience, it would be useful to examine whether some attribution types can lead to complete recovery in the reflexive stage. Examining concurrently the effects of attribution in the reflexive and reflective stages could serve to determine whether the attributions could have produced immediate relief, and would make it possible to directly examine changes in needs and negative affect over time.

### Conclusion

These three experiments provide empirical evidence for the role of attribution in moderating ostracism distress in the reflective stage. They also suggest which attribution types are the most effective in achieving relief and mitigating the negative effects of ostracism. They also pave the way for future empirical research on ways to better alleviate and recover from ostracism-related distress. Finally, death anxiety mediated the association between cuing an external attribution for ostracism on mood.

## Data Availability Statement

The raw data supporting the conclusions of this article will be made available by the authors, without undue reservation.

## Ethics Statement

The studies involving human participants were reviewed and approved by Ono Academic College review board. The patients/participants provided their written informed consent to participate in this study.

## Author Contributions

EY contributed to conception and design of the study, organized the database, performed the statistical analysis, wrote the first draft of the manuscript, and wrote sections of the manuscript.

## Conflict of Interest

The author declares that the research was conducted in the absence of any commercial or financial relationships that could be construed as a potential conflict of interest.

## Publisher’s Note

All claims expressed in this article are solely those of the authors and do not necessarily represent those of their affiliated organizations, or those of the publisher, the editors and the reviewers. Any product that may be evaluated in this article, or claim that may be made by its manufacturer, is not guaranteed or endorsed by the publisher.

## Figures and Tables

**Table:** 

For each question, please circle the number to the right that best represents the feelings you are experiencing RIGHT NOW.	Not at all				Extremely
Good	1	2	3	4	5
Bad	1	2	3	4	5
Friendly	1	2	3	4	5
Unfriendly	1	2	3	4	5
Angry	1	2	3	4	5
Pleasant	1	2	3	4	5
Happy	1	2	3	4	5
Sad	1	2	3	4	5

**Table:** 

For each question, please circle the number to the right that best represents the feelings you are experiencing RIGHT NOW.	Not at all				Extremely
**Belonging**					
I felt “disconnected” (R)	1	2	3	4	5
I felt rejected (R)	1	2	3	4	5
I felt like an outsider (R)	1	2	3	4	5
I felt I belonged to the group	1	2	3	4	5
I felt the other players interacted with me a lot	1	2	3	4	5
**Self esteem**					
I felt good about myself	1	2	3	4	5
My self-esteem was high	1	2	3	4	5
I felt liked	1	2	3	4	5
I felt insecure (R)	1	2	3	4	5
I felt satisfied	1	2	3	4	5
**Meaningful existence**					
I felt invisible (R)	1	2	3	4	5
I felt meaningless (R)	1	2	3	4	5
I felt nonexistent (R)	1	2	3	4	5
I felt important	1	2	3	4	5
I felt useful	1	2	3	4	5
**Control**					
I felt powerful	1	2	3	4	5
I felt I had control over the course of the game	1	2	3	4	5
I felt I had the ability to significantly alter events	1	2	3	4	5
I felt I was unable to influence the action of others (R)	1	2	3	4	5
I felt the other players decided everything (R)	1	2	3	4	5

## Footnotes

^1^The other scenarios that were examined in the pilot study in addition to those chose were: External – unstable attribution condition – “the computer was not working very well today” (Minternal = 3.83, SDinternal = 0.98; Mstable = 4.01, SDstable = 0.88) and “the program was not the final one that was uploaded” (Minternal = 3.92, SDinternal = 0.99; Mstable = 3.81, SDstable = 0.79); External – stable attribution condition – “This often happens during experiments in the lab” (Minternal = 3.14, SDinternal = 0.88; Mstable = 2.21, SDstable = 0.68) and “this often happens when three people are playing together” (Minternal = 3.30, SDinternal = 1.09; Mstable = 2.11, SDstable = 0.92); Internal – stable attribution condition – “This often happens when players differ in ability” (Minternal = 2.01, SDinternal = 0.86; Mstable = 2.17, SDstable = 0.82); and “this often happens when the player is not liked” (Minternal = 1.97, SDinternal = 0.85; Mstable = 2.22, SDstable = 0.77); Internal – unstable attribution condition – “I saw that you were not concentrating” (Minternal = 2.31, SDinternal = 1.00; Mstable = 4.02, SDstable = 0.90) and “This often happens when players do not practice” (Minternal = 2.19, SDinternal = 0.99; Mstable = 3.98, SDstable = 0.76).

^2^*It could be claimed that differences would be found between negative and positive emotions and that after an ostracism experience negative emotions would increase while positive emotions would remain unchanged. To control for this possibility, the same analyses were conducted separately for the positive and negative emotion scores. No differences were found, so that only the combined measure is reported*.
